# Addressing the gender-knowledge gap in glucose-6-phosphate dehydrogenase deficiency: challenges and opportunities

**DOI:** 10.1093/inthealth/ihy060

**Published:** 2018-09-03

**Authors:** Gonzalo J Domingo, Nicole Advani, Ari W Satyagraha, Carol H Sibley, Elizabeth Rowley, Michael Kalnoky, Jessica Cohen, Michael Parker, Maureen Kelley

**Affiliations:** 1 PATH, Seattle, WA, USA; 2 Eijkman Institute for Molecular Biology, Jakarta, Indonesia; 3 WorldWide Antimalarial Resistance Network, University of Washington, Seattle, WA, USA; 4 The Ethox Centre and Wellcome Centre for Ethics and Humanities, Nuffield Department of Population Health, University of Oxford, UK

**Keywords:** Chromosome, Gender, Glucose-6-phosphate dehydrogenase, Point-of-care, X-inactivation

## Abstract

Glucose-6-phosphate dehyrdgoenase (G6PD) deficiency is a common X-linked genetic trait, with an associated enzyme phenotype, whereby males are either G6PD deficient or normal, but females exhibit a broader range of G6PD deficiencies, ranging from severe deficiency to normal. Heterozygous females typically have intermediate G6PD activity. G6PD deficiency has implications for the safe treatment for *Plasmodium vivax* malaria. Individuals with this deficiency are at greater risk of serious adverse events following treatment with the only curative class of anti-malarials, 8-aminoquinolines, such as primaquine. Quantitative diagnostic tests for G6PD deficiency are complex and require sophisticated laboratories. The commonly used qualitative tests, do not discriminate intermediate G6PD activities. This has resulted in poor understanding of the epidemiology of G6PD activity in females and its corresponding treatment ramifications. New simple-to-use quantitative tests, and a momentum to eliminate malaria, create an opportunity to address this knowledge gap. While this will require additional resources for clinical studies, adequate operational research, and appropriate pharmacovigilance, the health benefits from this investment go beyond the immediate intervention for which the G6PD status is first diagnosed.

## G6PD deficiency

Glucose-6-phosphate dehydrogenase (G6PD) is a critical housekeeping enzyme in RBC that supports protective systems against oxidative challenge by producing the reduced form of nicotinamide adenine dinucleotide phosphate.^1,2^ G6PD deficiency is the most common human enzyme defect, affecting over 400 million people worldwide. The *g6pd* gene is a highly polymorphic human gene, with over 200 mutations identified.^3,4^ Red blood cells are especially vulnerable to the effects of these mutations because they cannot replenish their supplies of the enzyme once they mature and enter the bloodstream. As a result, they are susceptible to hemolysis when subjected to oxidative stress, induced by certain therapies, such as antimalarial 8-aminoquinolines, a few antibiotics and some anti-inflammatories. Hemolysis can also be activated by other exogenous agents, including foods (e.g., fava beans), henna and some infections (e.g., hepatitis A or B, pneumonia, typhoid fever).^1,5^ These hemolytic episodes can range from mild to life-threatening, depending on the variant of G6PD deficiency, the dose of the precipitating factor, age (severe reactions are more life-threatening in children) and coexisting morbidities. However, until one of the stressors is experienced, G6PD-deficient individuals may not even be aware of their condition. Severe hemolysis can lead to anemia, kidney damage and even death. In rural and low-income settings, lack of access to monitoring of symptoms and supportive care can further increase risk of morbidity and mortality.

**Figure 1.**
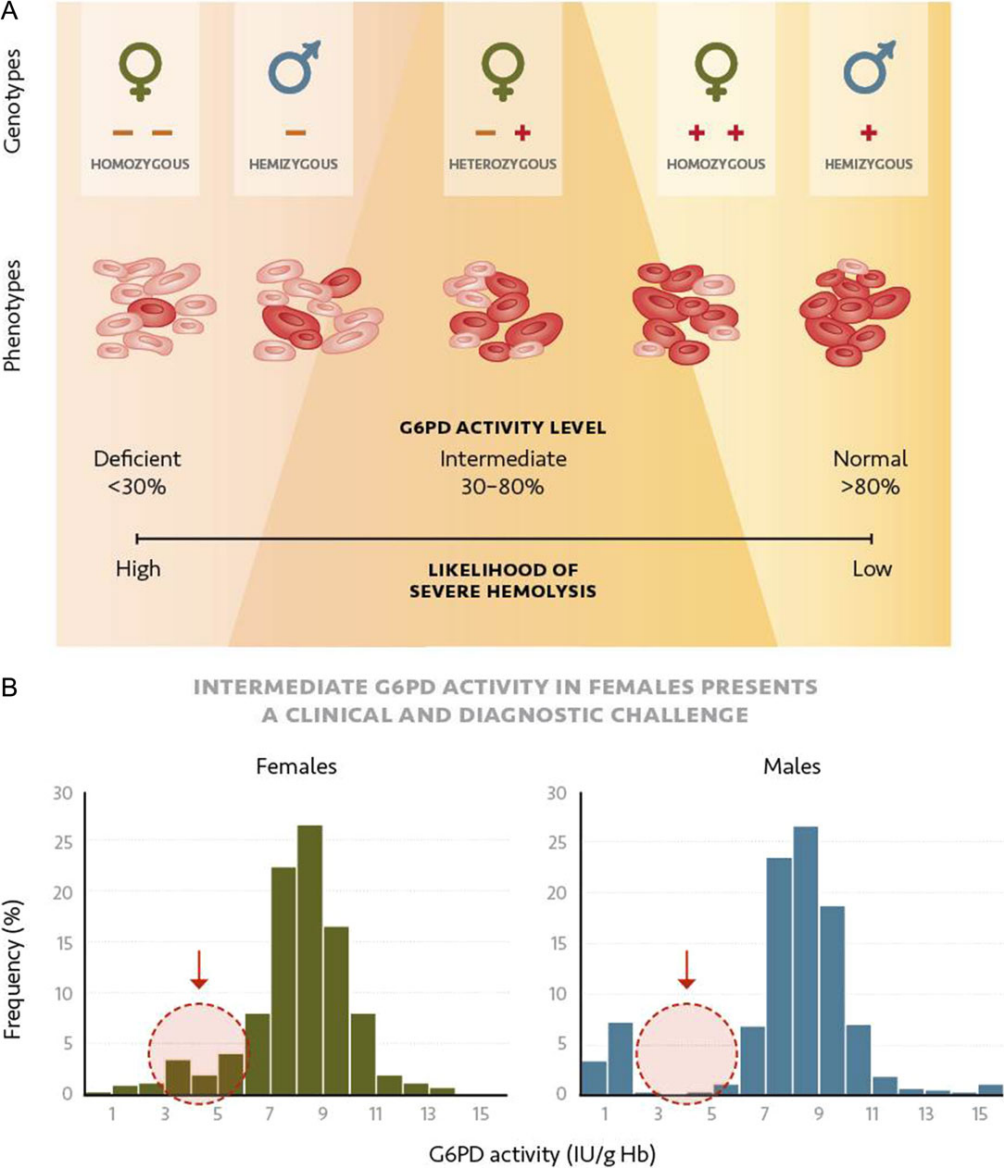
Association between G6PD genotype in males and females, and red blood cell G6PD activity levels in a population. Histograms show the distributions of hemoglobin-normalized G6PD activity levels for **(A)** males and **(B)** females.

The *g6pd* gene is located on the X chromosome, so females have two alleles and males have only one.^6^ To respond to this genomic imbalance, early in embryonic development in females, one X chromosome in each cell is inactivated. Consequently, males with a single X chromosome carry either a G6PD-deficient or G6PD-normal genotype, and females with two alleles can be homozygous or heterozygous for G6PD. In some cases, heterozygous females carry one allele encoding a G6PD enzyme with normal activity and one allele encoding an enzyme with G6PD-deficient activity (Table 1). As a result of random X chromosome inactivation, individual RBCs in heterozygous females express the G6PD enzyme from either one or the other allele, resulting in two RBC populations based on the *g6pd* allele expressed. The relative ratio of the two RBC populations determines the G6PD activity of the female. These ratios range from a high proportion of RBCs with the normal G6PD enzyme to a high proportion of RBCs with the deficient G6PD enzyme. The resulting overall levels of G6PD enzyme activity in heterozygous females mainly range from 30% to 80% of normal G6PD activity; values within this range are considered as intermediate (Figure 1).^6–11^

**Table 1.** G6PD genotypes and associated phenotypes. The *g6pd* gene lies on the X chromosome. Males have only one allele, encoding either a G6PD enzyme with deficient activity (Def.) or normal activity (Norm.). Females have two alleles so they can have either two identical alleles (homozygous) or two different alleles (heterozygous). The associated phenotypes are described in the right two columns

Genotype				Phenotype	
Male		Female		Category	% Normal activity^a^
Type	Allele	Type	Allele		
Hemizygous	Def.	Homozygous	Def._1_ Def._1_	Severe deficient	<30%
		Heterozygous	Def._1_ Def._2_		
		Heterozygous	Def. Norm.	Intermediate or mildly deficient	Mostly between 30% and 80%^b^
Hemizygous	Norm.	Heterozygous	Norm._1_ Norm._2_	Normal	>80%
		Homozygous	Norm._1_ Norm._1_		

^a^Normal activity or 100% can be defined as the median activity of male hemizygous normal.

^b^Heterozygous females can range from severely deficient G6PD levels to normal, but lie mostly within the 30–80% activity range.

Accurate screening and counseling for G6PD deficiency among females also has implications for newborn health outcomes. G6PD deficiency is often first manifested in newborns as jaundice, resulting from hyperbilirubinemia, which can lead to kernicterus, a form of brain damage, if unchecked.^12–14^ Although more than half of all newborns develop jaundice from various causes in the first week of life, there is a higher rate of hyperbilirubinemia in infants who are G6PD deficient than in G6PD-normal infants; among these deficient infants, the requirement for exchange transfusion is higher than among infants with jaundice due to other causes.^14^ In 1989, the WHO working group on G6PD deficiency recommended that ‘whenever possible, neonatal screening should be performed … in populations where G6PD deficiency is common (i.e., where it affects more than three to five percent of males).’^15^ To avert serious consequences, people in areas known to have a high prevalence of G6PD deficiency should have access to testing, either as newborn screening or later in life—for instance, before being treated with drugs that may precipitate a hemolytic episode.

## Diagnostics for G6PD deficiency

The gold standard for determining G6PD status is through direct measurement of G6PD activity, normalized either by red blood cell count or hemoglobin concentration.^16^ The status is then defined based on where this value lies relative to a normal G6PD value. Normal G6PD activity, or 100% activity, in a population can be defined by the median value of hemizygous males with a G6PD normal allele.^15–17^ Unfortunately, as a consequence of poor standardization across G6PD enzyme assay kits and the high sensibility of an enzyme assay to all conditions, including salts, pH and temperature, it is hard to attribute laboratory-to-laboratory variation in normal G6PD values to differences in the population sampled or inter-laboratory variability coupled with inter-assay variability.

Additionally, the current quantitative assays are challenging to implement in clinical laboratories. As a result, qualitative tests (such as fluorescent spot tests) are used most commonly to screen for this deficiency. A combination of the enzyme kinetics and the population G6PD genetics means that these qualitative tests can be formulated to provide a robust discriminatory threshold at 30–40% of normal G6PD activity.^8,9,18^ This enzyme activity threshold allows the tests to accurately identify all hemizygous males with a G6PD-deficient allele and females with two G6PD-deficient alleles, but heterozygous females can only be discriminated from normal activity if less than 20% of their RBCs express the normal G6PD alleles.^7,8,10^

Microscopy or flow cytometry-based assays that determine G6PD levels in individual RBCs are extremely informative for understanding the mosaic expression of *g6pd* alleles in individual heterozygous females.^7,9,10,19,20^ These are not practical clinical assays, however. Likewise, genetic tests are deterministic regarding an individual’s G6PD genotype, but are not clinically useful for understanding the phenotypic (clinically relevant) G6PD status of heterozygous females with one normal and one deficient G6PD allele.

## Population distributions of G6PD activity

**Figure 2.**
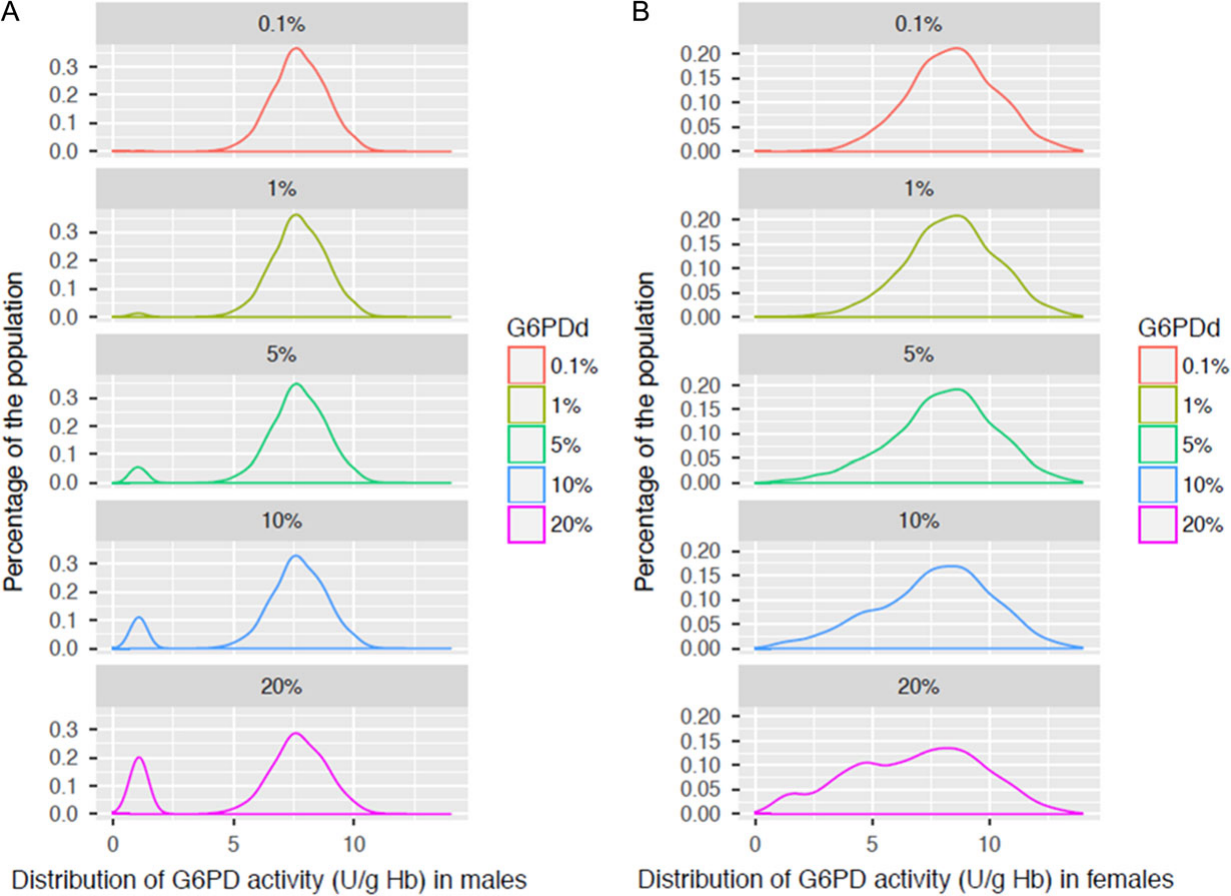
Population distribution for males and females arranged by individual G6PD activity level (U/g Hb) at different G6PD-deficient allele frequencies in males. The distributions were modeled based on the Hardy–Weinberg equilibrium and using empirical data from a cross-sectional G6PD study, whereby G6PD activity was measured by the Trinity quantitative test (G-6-PDH 35-A).^46^ Population distributions are shown for **(A)** males and **(B)** females. These distributions were used for Table 2.

The population distribution of G6PD activity can be described at a genetic level through the Hardy–Weinberg equilibrium, which can be used to predict the genotype distributions for two alleles in a population.^21^ From a clinical perspective, however, it is the phenotype distribution that determines the probable proportion of the population that is G6PD deficient (or below a given G6PD activity threshold). Although many publications describe either the genotypic or the phenotypic distribution, there are few comprehensive data sets that include both sets of data. Because males are both phenotypically and genotypically either G6PD deficient or normal, it is most reliable to express G6PD deficiency prevalence based on the male G6PD deficiency prevalence. Based on data sets for which both phenotypic and genotypic data are available in combination with the Hardy-Weinberg equilibrium for two distinct alleles, it is possible to generate a model that generates G6PD activity population profiles by sex (Figure 2).

The prevalence of G6PD deficiency and predominant G6PD deficient variants can vary by ethnicity.^2,3,22^ This prevalence has an impact on the population G6PD activity distributions, especially for females (Figure 2). From these profiles, estimates of the relative proportion of males and females that lie under any given threshold can be derived (Table 2). The severity of the underlying prevalent *g6pd*-deficient allele affects the population distribution for the G6PD activity levels of heterozygous females (e.g., leading to female populations being skewed toward higher or lower G6PD activity levels), but it does not significantly impact the male distributions.^20^

**Table 2.** Representation of males and females defined as G6PD deficient, assuming different thresholds for deficiency. The prevalence of G6PD deficiency is expressed in terms of hemizygous males with a G6PD-deficient allele. The numbers are calculated for a population of 10,000 with equal male and female distribution

Male G6PD deficiency prevalence		Threshold G6PD activity expressed as percent of normal														
		30%			40%			60%			70%			80%		
		M	F	T	M	F	T	M	F	T	M	F	T	M	F	T
0.1%	No.	5	1	6	5	12	17	22	117	139	106	285	391	417	578	995
	% def.	83	17	100	29	71	100	16	84	100	27	73	100	42	58	100
	% pop.	0.1	0.0	0.1	0.1	0.2	0.2	0.4	2.3	1.4	2.1	5.7	3.9	8.3	11.6	10.0
1%	No.	50	8	58	50	32	82	67	167	234	151	349	500	458	652	1110
	% def.	86	14	100	61	39	100	29	71	100	30	70	100	41	59	100
	% pop.	1.0	0.2	0.6	1.0	0.6	0.8	1.3	3.3	2.3	3.0	7.0	5.0	9.2	13.0	11.1
5%	No.	250	54	304	250	124	374	267	379	646	350	619	969	639	952	1591
	% def.	82	18	100	67	33	100	41	59	100	36	64	100	40	60	100
	% pop.	5.0	1.1	3.0	5.0	2.5	3.7	5.3	7.6	6.5	7.0	12.4	9.7	12.8	19.0	15.9
10%	No.	500	124	624	500	237	737	517	656	1173	595	968	1563	864	1313	2177
	% def.	80	20	100	68	32	100	44	56	100	38	62	100	40	60	100
	% pop.	10.0	2.5	6.2	10.0	4.7	7.4	10.3	13.1	11.7	11.9	19.4	15.6	17.3	26.3	21.8
20%	No.	1000	335	1335	1000	513	1513	1016	1191	2207	1086	1608	2694	1328	1984	3312
	% def.	75	25	100	66	34	100	46	54	100	40	60	100	40	60	100
	% pop.	20.0	6.7	13.4	20.0	10.3	15.1	20.3	23.8	22.1	21.7	32.2	26.9	26.6	39.7	33.1

For each prevalence, the total number (No.) of males (M), females (F), and the sum of the two (T) that have less than the threshold G6PD activity levels are given.

The relative proportions of the two genders from the total number of deficient (% def.) as well as the proportion (% pop.) of all males, all females, and the total population are also given.

The distributions show that deficient males predominantly lie under the 30% G6PD activity thresholds and heterozygous females contribute predominantly to the intermediate activity ranges of less than 80% activity.

## Malaria treatment and G6PD deficiency

G6PD deficiency and malaria intersect in two very different ways. Epidemiologically, it is striking that the G6PD prevalence and malaria prevalence maps overlap. Globally, while G6PD-deficiency prevalence ranges from 0% up to 20%, the mean prevalence in malaria-endemic populations is 8%.^22^ From a biological perspective, this is remarkably similar to the pattern of the mutant β-hemoglobin gene that causes sickle cell anemia when homozygous. In both cases, these deleterious genes appear to confer protection from severe malaria.^23–25^

From a case-management perspective, antimalarial drugs and scientific awareness of G6PD deficiency have a long history, starting from early trials of the curative drug for *Plasmodium vivax* malaria, primaquine, which led to the initial identification of G6PD deficiency and its association to hemolysis.^26^ As an 8-aminoquinoline, primaquine should not be prescribed to patients with G6PD deficiency. An antimalarial drug called Lapdap (chlorproguanil-dapsone) had to be removed from the market after launch due to unacceptable numbers of adverse events in Africa, resulting from exposure of G6PD-deficient patients with malaria to the oxidative drug component dapsone.^27^

Antimalarial drugs that target the blood-stage malaria parasites (schizonts) cure patients of *Plasmodium falciparum* malaria, but not *P. vivax* malaria. This is because *P. vivax* parasites can remain dormant in the liver as hypnozoites, which typical antimalarial drugs cannot reach. Thus, *P. vivax* patients who have only been treated with anti-schizonticidal drugs are likely to relapse from the same infection weeks or months later. These relapses result in incremental morbidity in the form of progressively more severe anemia and, in vulnerable individuals, an overall risk of mortality similar to that of *P. falciparum* malaria.^28,29^

From a disease-burden perspective at a community level, each relapse represents an opportunity for onward infection, particularly for *P. vivax*, in which the sexual gametocytes required for the vector appear early during the blood-stage reinfection. Relapse can contribute to over 75% of disease in a community.^30,31^

The only drugs known to cure *P. vivax* infection are 8-aminoquinoline based. Primaquine, which has been available since the 1950s, is administered as a 14-d regimen at 0.25–0.50 mg base/kg body weight daily or as a 7-d regimen at 0.50 mg base/kg body weight or once a week for 8 wk at 0.75 mg base/kg. An investigational drug, tafenoquine, also an 8-aminoquinoline, with similar safety considerations for G6PD deficiency, has completed phase 3 clinical trials. Tafenoquine, in contrast to primaquine, requires only a single dose regimen.^32^ A single dose of primaquine (0.25 mg base/kg) is also used to kill gametocytes in an effort to block onward transmission, although the dose is thought to be low enough to be safe, even for G6PD-deficient subjects.^33^

For a range of reasons, policy and practice around primaquine prescription and G6PD deficiency have not been very consistent.^26^ Perhaps driven by the fact that primaquine treatment can be interrupted at any time, coupled with poor awareness of the G6PD-deficiency prevalence in many malaria-endemic populations and poor pharmacovigilance, national treatment guidelines have not always required G6PD testing of a patient before administering the drug, even in countries where there is a significant prevalence of G6PD deficiency. By contrast, in other countries, such as Malaysia and Lao, knowledge of a patient’s G6PD status is an absolute requirement before prescribing primaquine. In 2015, the WHO provided stronger recommendations with respect to testing for G6PD deficiency and administration of high-dose primaquine.^34^ Despite being available for over 60 y, primaquine is widely underused due to the concern of its reactions with G6PD deficiencies, its 14-d regimen raising adherence issues, and perhaps also an underappreciation of the impact of relapse on the patient, as well as on transmission.

The importance of being able to determine the G6PD status of a patient with *P. vivax* has risen in recent years with increasing awareness of the contribution of relapse to disease, increasing awareness of the risk associated with G6PD deficiency and primaquine, and potential availability of the single-dose cure for *P. vivax*, tafenoquine. The clinical trials for tafenoquine set a threshold of 70% G6PD activity for eligibility for receiving tafenoquine to ensure females with intermediate G6PD deficiency would not receive the treatment, given the largely unknown risk of clinically significant hemolysis. Interestingly, from 70% to 80% there is a significant increase in males that lie under the threshold, all of which are likely to be G6PD normal, but they fall in that range because of how 100% activity is defined (Figure 2 and Table 2). If approved for use, tafenoquine would require testing for G6PD deficiency prior to prescription; only the advent of new, easy-to-use, quantitative diagnostic tests for G6PD deficiency will allow this requirement to be managed at the clinic level and the benefit from this new single-dose regimen for *P. vivax* to be realized.

## Risk associated with degree of G6PD deficiency

The risk of hemolysis increases with increasing drug dose and decreasing G6PD levels in a patient’s blood.^26^ The particular genetic G6PD-deficient trait can influence the ability of a patient to recover from drug exposure. The WHO definitions for severity of G6PD deficiency primarily categorize hemizygous G6PD-deficient males, as well as women homozygous for G6PD-deficient alleles, as G6PD deficient (less than 30% of normal); most heterozygous women are included in the intermediate G6PD activity range of 30–80% normal; above 80% is considered to be normal G6PD activity. Other concurrent blood disorders may also contribute to the red blood cell susceptibility.

These categories inform clinical management of G6PD deficiency, whereby people are not prescribed drugs for which G6PD deficiency is a safety concern if they are deficient (less than 30%). This threshold is reinforced by the fact that qualitative tests tend to categorize patients as G6PD deficient at approximately this level. The fluorescent spot test (FST), which is most commonly used in clinical settings, may be used to also categorize women with less than 40% activity as G6PD deficient if spots with intermediate signal are interpreted as deficient.

In the case of drug-associated risk of hemolysis, there is surprisingly little data to inform thresholds for safety or to challenge the assumption that G6PD deficiency greater than 30% is safe. This paucity of data is particularly relevant to females. Very few studies have looked at drug safety in females with intermediate G6PD activity. For primaquine, the perception of safety for females with intermediate activity greater than 30% of normal is reinforced by years of clinical practice, using the FST in settings with little pharmacovigilance or follow-up, creating a gender inequity in safety data. Recent studies seeking to address this knowledge gap at minimum suggest that the use of qualitative tests in women may not be adequate for case management with primaquine. They also indicate the absolute need to generate more safety data for women.^35–37^

The discussion on females with intermediate G6PD activity and drug-associated risk is also conflated with the notion that G6PD-deficient people are less likely to present with severe malaria symptoms, as G6PD deficiency reduces the severity of malaria, and that females with intermediate G6PD deficiency and malaria are rarely seen. This perception is likely to be biased by both the poor sensitivity of malaria rapid diagnostic tests (RDTs), particularly *P. vivax* RDTs, and the use (when used) of qualitative tests for G6PD deficiency that would not identify females with intermediate activities.

In newborn G6PD screening, there is also increasing recognition that the 30% threshold misses female newborns with high risk of progressing to severe hyperbilirubinemia, with high reticulocyte count possibly contributing to this. In one study, the authors recommend replacing the FST as the screening assay by a quantitative assay, as this allows increasing the threshold defining G6PD deficiency to improve detection rates for infants at high risk earlier, allowing closer monitoring.^38^ Increasing the thresholds for easier identification of female newborns at risk of developing severe hyperbilirubinemia has also been suggested elsewhere.^39–43^

## Improved testing for G6PD deficiency: quantitative diagnosis for reducing the gender bias

Due to frequently inadequate or inaccurate information about the true prevalence of G6PD deficiency in women, healthcare providers may perceive G6PD deficiency as having little or no impact on females. This gender-biased health misconception can have severe health impacts on these patients. It can also be one of the reasons that health systems do not prioritize the testing of G6PD deficiency over competing health priorities.

Currently, G6PD testing is available only in some communities where there is a high prevalence of G6PD deficiency.^1^ When it is done, it is primarily through a qualitative test that underestimates G6PD deficiency in women. In the absence of more routine quantitative testing for G6PD deficiency and more robust pharmacovigilance, the knowledge gap in risk of hemolysis between males and females will not be narrowed. This knowledge gap has been extremely challenging to address operationally, especially in malaria-endemic settings, due to the complexity of performing quantitative G6PD testing. With the advent of point-of-care quantitative tests for G6PD deficiency, more accurate routine testing may become feasible, initially in the context of clinical trials and operational studies, and subsequently in healthcare settings with high *P. vivax* malaria transmission. Most immediately, these new quantitative diagnostic tests will be required for use with tafenoquine to ensure its safe use—especially in women—but the potential of a simple-to-use quantitative test for G6PD will likely be relevant for future drugs with an associated G6PD risk.

Furthermore, the failure to identify females with heterozygous G6PD normal/deficient alleles and the highest likelihood of intermediate enzyme activity (30–80% of normal) goes beyond access to best-treatment options for the individual woman. It represents a lost opportunity to identify relatives with this genetic condition. Women heterozygous for G6PD deficiency with intermediate activity levels are approximately twice as prevalent in a population than homozygous G6PD-deficient females and hemizygous G6PD-deficient males (Table 2). Many patients who learn their status will encourage their family members to get tested, which encourages better, earlier, and more sustained management of the condition across families and communities. It also has implications for newborn screening and possible health implications, as noted above.

## Resource and funding implications of increased access to accurate G6PD status diagnosis

As new point-of-care tests for G6PD deficiency become available and countries seek to safely increase access to *P. vivax* radical cure with new malaria treatments, such as tafenoquine, malaria programs and health systems have an opportunity to address the existing gender gap associated with accurate measurement of G6PD deficiency and associated drug-related adverse events among women. Although it will require additional resources and concerted planning, this is an important opportunity for which the malaria community should be prepared.^44^

The potential for widespread introduction of these new tests raises important questions about health systems’ preparedness for handling the genetic test results. While the WHO has established clear guidelines on how to manage malaria cases in conjunction with G6PD testing, there is less clarity on how to manage the genetic counseling dimension of this condition. Genetic counseling messages and materials need to be informative and actionable, based on the settings where these tests will be used, while also taking into consideration the broader implications of these test results beyond malaria treatment. As there is little guidance on whether or how to encourage G6PD testing among a woman’s relatives following her G6PD diagnosis, or how best to guide monitoring of infants of G6PD-deficient mothers for signs of a hemolytic event, these issues should also be considered as part of the broader G6PD introduction planning. There is extensive experience with the return of genetic results from other health areas, and the malaria field can draw on these lessons, especially as they pertain to low-resource and low-literacy settings where G6PD deficiency is most prevalent and where access to care for conditions beyond malaria case management may be more limited. Investments in this area offer an opportunity to increase the cost-effectiveness of introducing an accurate, point-of-care G6PD test beyond the initial indication for which it was developed, benefiting not just the individual tested, but also the broader community.^45^

## Conclusions

As a consequence of the poor feasibility of performing quantitative testing for G6PD deficiency as part of malaria clinical management to date, G6PD-deficiency-associated risk in females is poorly understood and perhaps underestimated. Current national malaria-treatment guidelines and patient-management practices for women are supported by extremely limited data with regard to drugs for which G6PD deficiency is a safety concern. The qualitative tests most commonly used to check for G6PD deficiency in clinical settings are adequate for identifying males with G6PD deficiency, thus informing appropriate treatment options. However, these tests do not accurately define G6PD activity in females, potentially exposing women with intermediate G6PD activity to the risk of severe anemia, hemolysis and other health impacts.

Progress in the development of a new *P. vivax* antimalarial drug, tafenoquine, has driven the development of simple and reliable quantitative tests for G6PD deficiency. It will now become operationally easier to identify women and girls across the broad range of G6PD deficiency, collect safety data associated with females with intermediate G6PD activity and, in turn, provide appropriate treatment for these patients. With adequate funding, research and pharmacovigilance, these efforts can be realized and can improve gender equity in the safe and effective delivery of treatments for both males and females, particularly in low-income, malaria-endemic areas, where both malaria and G6PD deficiencies have the greatest impact on women and their families. Beyond malaria, G6PD status provides important clinical information for other health conditions that may impact this target group. The timeliness of addressing these issues—both within broader malaria strategic initiatives and at the national level within health system-strengthening efforts associated with the introduction of these new drugs and diagnostics—will help ensure the greatest potential health benefit is realized among women and their families. Furthermore, these efforts will help minimize the long-standing gender-disparity in G6PD-deficiency data, directly informing and improving malaria treatment strategies worldwide.
